# Supplementary figures and images for: High-Throughput Phenotyping Accelerates the Dissection of the Phenotypic Variation and Genetic Architecture of Shank Vascular Bundles in Maize (Zea mays L.)
Source: Plants (Basel). 2022 May 18;11(10):1339. doi: 10.3390/plants11101339 (PMC9145235; doi:10.3390/plants11101339)

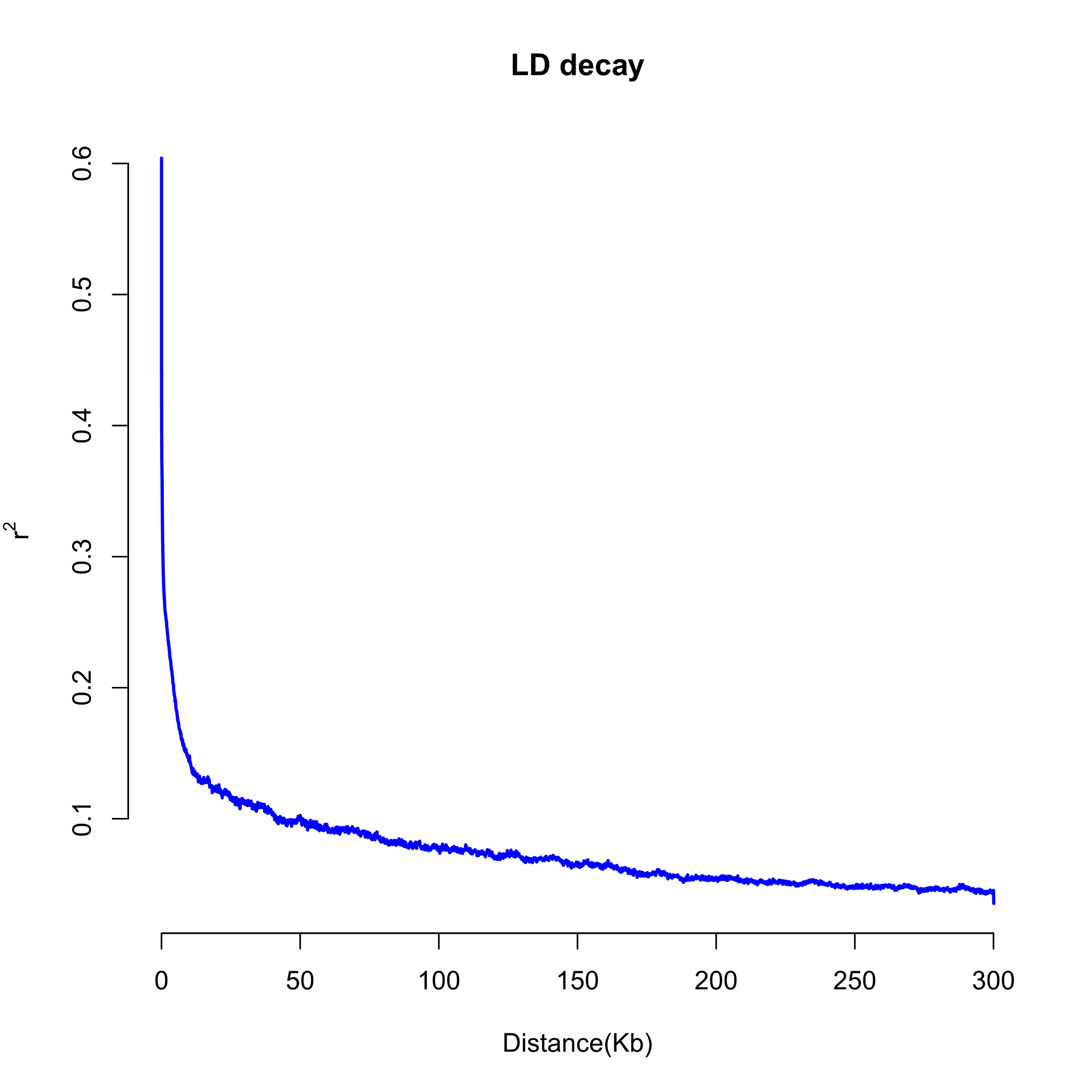

Supplement: Supplementary file 1 [file plants-11-01339-s001.zip › Supplementary Figure S1.png]

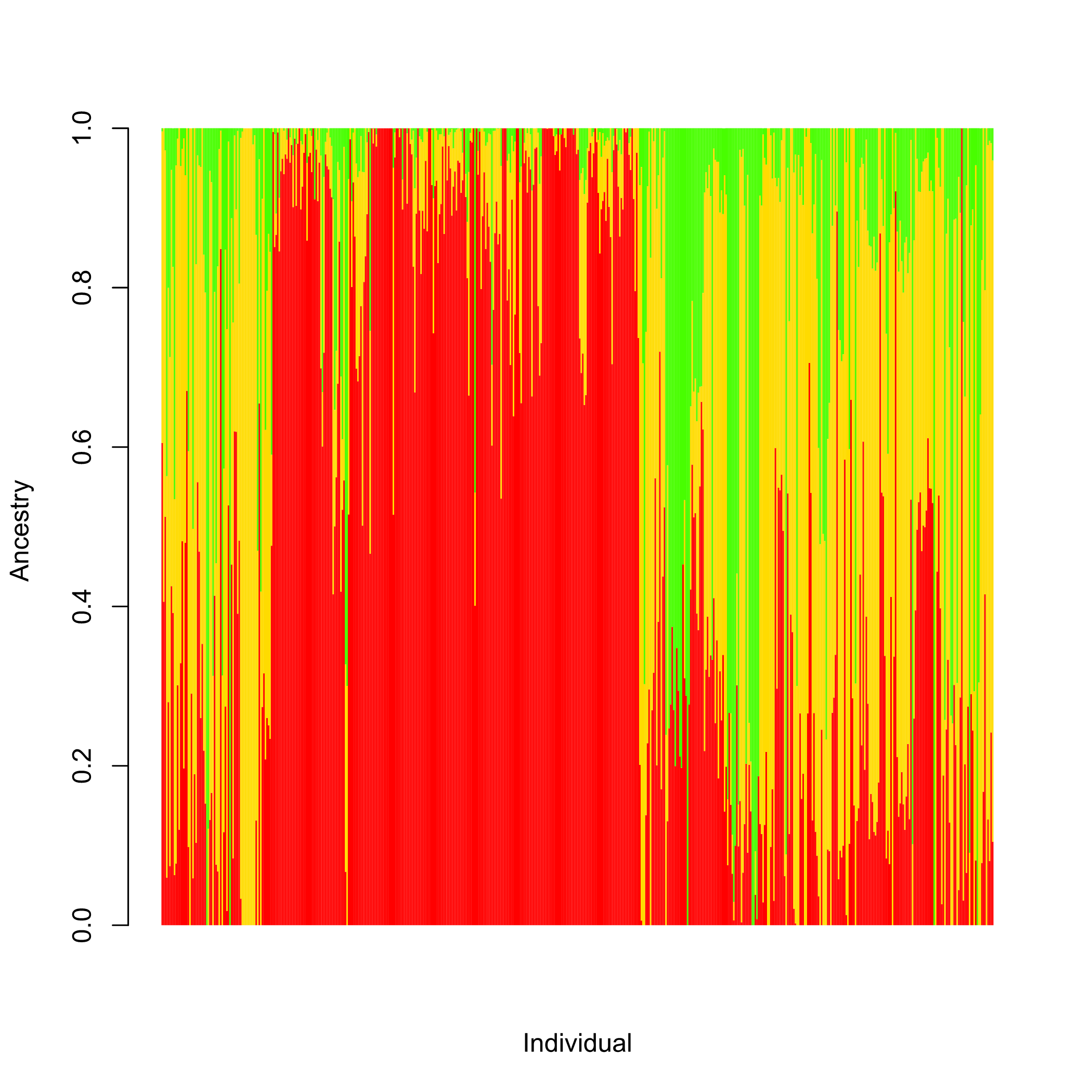

Supplement: Supplementary file 1 [file plants-11-01339-s001.zip › Supplementary Figure S2.png]
